# Supplementary material for: Suicidal ideation and interrelated psychiatric disturbances in rheumatoid arthritis: Evidence from a Vietnamese cohort
Source: PLoS One. 2026 Mar 9;21(3):e0342909. doi: 10.1371/journal.pone.0342909 (PMC12970863; doi:10.1371/journal.pone.0342909)
Supplement: S1 Table — (DOCX) [file pone.0342909.s001.docx]

Table S1. Preliminary validation metrics of psychometric scales in the study cohort

| **Scale** | **Cronbach's alpha** | **Item–Item Corr** | **Item–Total Corr** | **KMO (Overall)** | **CFA** |
| --- | --- | --- | --- | --- | --- |
| PHQ-9 | 0.92 | 0.279÷0.733 | 0.512÷0.828 | KMO=0.89; Bartlett p<0.001 | 1-factor model: χ²=80.818, df=27, CFI=0.926, RMSEA=0.129 |
| HAM-A | 0.93 | 0.158÷0.769 | 0.541÷0.766 | KMO=0.89; Bartlett p<0.001 | 2-factor (Psychic vs Somatic): χ²=241.7, df=64, CFI=0.823, RMSEA=0.152 |
| ISI | 0.96 | 0.734÷0.926 | 0.879÷0.927 | KMO=0.91; Bartlett p<0.001 | 1-factor model: χ²=105.1, df=14, CFI=0.915, RMSEA=0.233 |
| ASEX | 0.96 | 0.834÷1.000 | 0.883÷0.981 | KMO=0.5 (limited sample, elderly & severe patients, low sexual activity frequency); CFA not performed | NA |
| EQ-5D-5L | 0.86 | 0.376÷0.837 | NA | NA | NA |
