# Supplementary material for: Suicidal ideation and interrelated psychiatric disturbances in rheumatoid arthritis: Evidence from a Vietnamese cohort
Source: PLoS One. 2026 Mar 9;21(3):e0342909. doi: 10.1371/journal.pone.0342909 (PMC12970863; doi:10.1371/journal.pone.0342909)
Supplement: S2 Table — (DOCX) [file pone.0342909.s002.docx]

Table S2. Structural equation modeling (SEM) fit indices, standardized loadings, and diagnostics

| Component | Results |
| --- | --- |
| Model fit | χ² = 0.002, df = 1, p = 0.964; CFI = 1.00; TLI = 1.02; RMSEA = 0.000 (90% CI: 0–0); SRMR = 0.000 |
| R² | DEP = 0.855; ANX = 0.631; SEX = 0.080; SLP = 0.792 |
| Distribution diagnostics | All indicators showed acceptable skewness (0.01–0.98) and kurtosis (–1.02 to 2.44) for MLR estimation |
| Multicollinearity | VIF values ranged 1.07–1.89 (all below common thresholds), indicating no problematic multicollinearity |
| Modification indices | Max MI = 0, suggesting no model modifications required |
