# Supplementary figures and images for: Suicidal ideation and interrelated psychiatric disturbances in rheumatoid arthritis: Evidence from a Vietnamese cohort
Source: PLoS One. 2026 Mar 9;21(3):e0342909. doi: 10.1371/journal.pone.0342909 (PMC12970863; doi:10.1371/journal.pone.0342909)

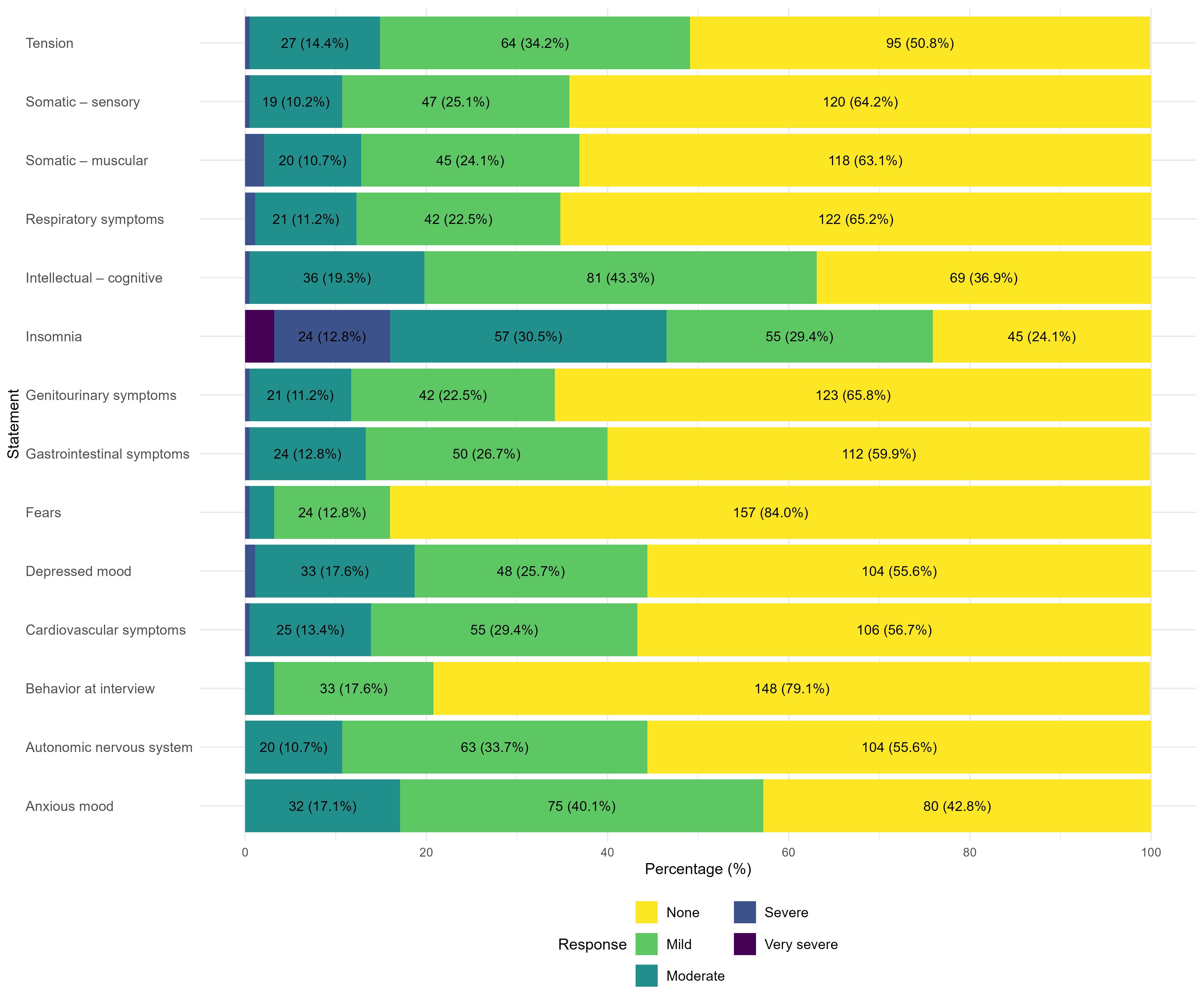

Supplement: S2 Fig — (TIFF) [file pone.0342909.s004.tiff]
