# Supplementary material for: Suicidal ideation and interrelated psychiatric disturbances in rheumatoid arthritis: Evidence from a Vietnamese cohort
Source: PLoS One. 2026 Mar 9;21(3):e0342909. doi: 10.1371/journal.pone.0342909 (PMC12970863; doi:10.1371/journal.pone.0342909)
Supplement: Attachment — Submitted filename: rasuicide_response letter.docx [file pone.0342909.s011.docx]

Ha Thi Thu Tran MD

Department of Psychiatry, Hanoi Medical University

1 Ton That Tung, Trung Tu, Dong Da, Hanoi, Vietnam

Phone: +84 904779798

Email: [tran_thuha@hmu.edu.vn](mailto:tran_thuha@hmu.edu.vn)

Professor Marco Innamorati

Editorial board

Plos One

November 7^th^, 2025

Subject: Revision and resubmission of manuscript ID PONE-D-25-50900

Professor Marco Innamorati

Thank you for giving us the opportunity to submit a revised draft of my manuscript titled ***“Suicidal ideation, mental health disturbances, and quality of life in rheumatoid arthritis: evidence from a Vietnamese cohort "*** to the Plos One Journal. The suggestions offered by the reviewers have been immensely helpful, and we also appreciate your insightful comments on revising the abstract and other aspects of the paper. We have included the reviewer comments immediately after this letter and responded to them individually, indicating exactly how we addressed each concern or problem and describing the changes we have made. The revisions have been approved by all authors. The changes have been highlighted within the manuscript, and the revised manuscript is attached to this email message.

We would like to thank the reviewers again for taking the time to review our manuscript. We look forward to hearing from you in due time regarding our submission and to respond to any further questions and comments you may have.

**Responses to Comments**

# **Reviewer 1:**

***Comment 1:***

Temporal Inconsistency (Abstract/Methods): A significant discrepancy exists between the study period cited in the Abstract ("December 2023 to September 2024") and the Methods section ("June 2024 to December 2024"). The authors must verify and state a single, accurate study period throughout the entire manuscript (Abstract and Methods).

***Response:***

Thank you for pointing out this inconsistency. We apologize for the oversight. The correct study period is June 2024 to December 2024. We have corrected this throughout the manuscript (Abstract, Methods, and any other instances) so the dates are consistent. The revised sentences are provided below and have been implemented in the manuscript.

***Comment 2:***

Line 136: The term "conventional sampling" is unscientific. Given the setting, the method should be correctly designated as "convenience sampling" or "consecutive sampling." Please correct the nomenclature.
***Response:***

We appreciate the reviewer’s clarification. The correct sampling method used in this study was consecutive sampling, and the terminology has been corrected accordingly in the Methods section.

***Comment 3:***

For each psychometric instrument used (MADRS, HAM-A, ISI, ASEX, EQ-5D-5L), please specify the instrument type (e.g., clinician-administered rating scale, self-report questionnaire) to clearly delineate the data source.
***Response:***

All psychometric assessments in our study—including the MADRS, HAM-A, ISI, ASEX, and EQ-5D-5L—were clinician-administered rating scales, and we have now explicitly stated this for all the instruments in the Methods section.

***Comment 4:***

To establish the reliability of the measurements in this specific cohort, please provide the Cronbach's alpha value for each instrument as calculated in this study, or cite the Alpha from the Vietnamese validation study if not recalculated.
***Response:***

Thank you for this helpful suggestion. In response, we have added a summary of internal consistency results for all psychometric instruments in Supplementary Table S1. Cronbach’s alpha was recalculated for all scales in the present cohort to confirm measurement reliability. The obtained values were as follows: HAM-A = 0.93, ISI = 0.96, ASEX = 0.86, and EQ-5D-5L = 0.86. The MADRS had been previously validated and culturally adapted in a separate Vietnamese study by our team, which reported good internal consistency (Cronbach’s alpha = 0.93). Corresponding text describing these results has been added to the Study outcomes section

***Comment 5:***

Line 94: The sentence "Anxiety were assessed..." contains an error in subject-verb agreement. Correct to "Anxiety was assessed..."

***Response***:

Thank you for pointing this out. The sentence has been corrected to read: “Anxiety was assessed using the Hamilton Anxiety Rating Scale (HAM-A).”

***Comment 6:***

Line 132: Since suicidal ideation was assessed via structured interviews, it represents a clinically determined prevalence. Please explicitly state in the Methods that this is a direct or clinically determined prevalence rather than an indirect estimate based on a screening questionnaire, as this is a key strength.

***Response:***

We appreciate the reviewer’s comment. As described in the Study Outcomes section under “Primary outcome,” suicidal ideation was assessed through direct clinical evaluation conducted by board-certified psychiatrists during structured interviews, based on established psychiatric diagnostic guidelines. Therefore, the reported prevalence represents a clinically determined prevalence, rather than an indirect estimate from a screening questionnaire. We have ensured this distinction is explicitly stated in the Methods section.

***Comment 7:***

Line 141: Greater detail is required for the clinical assessments:

oMorning Stiffness: Specify the measurement method for the duration of morning stiffness (i.e., whether it was patient self-reported in minutes or hours).

oAdverse Effects: Detail the method used to quantify or record "adverse effects" (e.g., chart review, specific patient interview, patient self-report).

***Response:***

We have now clarified that morning stiffness was measured via patient self-report in minutes. Similarly, adverse effects were captured using both structured patient interviews and chart review, ensuring comprehensive and standardized recording of any medication-related events. These details are now explicitly described in the Methods section.

***Comment 8:***

The manuscript inconsistently uses both "adverse effects" and "adverse events" related to pharmacological treatment. Please select one term ("adverse effects" is preferred in a clinical setting) and use it uniformly throughout the text. Ensure the acronym AEs is defined at its first appearance (e.g., "adverse effects (AEs)").

***Response:***

We have revised the manuscript to use the term “adverse effects (AEs)” consistently throughout the text. The acronym AEs is now defined at its first occurrence. No instances of “adverse events” remain in the revised manuscript.

***Comment 9:***

Line 220-221: The phrase "with a notable but imprecise estimate for suicidal ideation" is vague. Please rephrase this sentence to clearly explain what constitutes "imprecise" (e.g., lower statistical power, wider confidence interval, or smaller effect size compared to other outcomes).

***Response:***

We have revised the sentence to remove the vague phrasing and now report the associations clearly and directly using the corresponding effect estimates. The revised sentence reads: *“Female gender was associated with higher risks of depression (OR 3.80, 95% CI: 1.41–11.8) and anxiety (OR 3.82, 95% CI: 1.43–11.7).”*

***Comment 10:***

Line 312-313: The use of the term "prevalent" in this specific context is reportedly confusing. Please rephrase the sentence to use a clearer synonym like "frequent," "common," or "high-prevalence."

***Response:***

We agree that the use of the term “prevalent” in the original sentence could lead to ambiguity. To improve clarity and avoid epidemiological misinterpretation, we have revised the sentence to: *“This study demonstrates that suicidal ideation and other psychiatric disturbances are commonly observed among Vietnamese patients with RA.”*

**Reviewer 2**

***Comment 1***:

The abstract presents a coherent overview of the study but lacks clarity and interpretive balance in the results section. While the objectives and methods are adequately described, the results are presented as a list of prevalence figures and correlation coefficients without contextual meaning. The reader cannot easily grasp the magnitude or implications of the findings; for instance, the EQ-5D-5L mean score (0.6 ± 0.2) is reported without interpretation, leaving unclear whether this reflects a mild, moderate, or severe impairment in quality of life. The same issue applies to the reporting of psychiatric comorbidities, which would benefit from a brief narrative statement (e.g., indicating that depressive and anxiety symptoms were common and closely interrelated).

***Response:***

We agree that the results section of the abstract previously presented numbers without sufficient narrative interpretation, which limited clarity. In response, we have revised the abstract to:

- Provide a brief interpretive context for major findings (e.g., clarifying that an EQ-5D-5L score of 0.6 reflects substantial QoL impairment).
- Summarize psychiatric comorbidities using concise descriptive statements (e.g., “depressive, anxious, and insomnia symptoms were highly common and strongly interrelated”).
- Improve readability by integrating key results into a narrative rather than listing prevalence values and coefficients.

We believe these revisions enhance the interpretive balance and accessibility of the abstract while maintaining scientific precision.

***Comment 2***:

Overall, the introduction is rather limited and lacks a clear justification of the study rationale. It does not sufficiently explain the theoretical or empirical basis for the associations tested between suicidal ideation, psychiatric disturbances, and quality of life. This section should be expanded to clarify why these relationships were examined and how the study contributes new evidence to the field.

1.The introduction would benefit from a stronger articulation of the research gap. The authors should specify how their study adds to the limited literature on suicidal ideation in RA beyond reporting prevalence.

2.Clarify the distinction between primary and secondary study aims earlier for coherence.

***Response:***

We have revised the Introduction to clearly articulate the research gap and justify the study rationale. Specifically:

1. We emphasize that while psychiatric comorbidities such as depression, anxiety, insomnia, and sexual dysfunction are recognized in RA, direct evidence on suicidal ideation remains limited, particularly in the Vietnamese RA population. Prior studies often focus only on depression or anxiety, without systematically assessing suicidal thoughts or intent.
2. We explicitly distinguish the primary aim (describing prevalence and associated factors of suicidal ideation) from the secondary aim (providing an integrated overview of other psychiatric disturbances and their relationship with quality of life).
3. The rationale for examining these associations is now clearly presented: understanding suicidal ideation in the context of other psychiatric symptoms and QoL can inform early risk identification, guide integrated mental health care, and improve patient-centered outcomes in RA.
4. We have strengthened the narrative to show how this study contributes new evidence to the field by situating suicidal ideation as the central outcome and evaluating its interactions with broader mental health and behavioral symptoms.

These revisions address the reviewer’s concerns by providing a more coherent and theoretically grounded rationale for the study.

***Comment 3***:

The description of each psychometric scale (MADRS, HAM-A, ISI, ASEX) is overly extensive and reads more like a manual than a methods section.

***Response:***

We have shortened the descriptions of MADRS, HAM-A, ISI, and ASEX to avoid manual-like detail while retaining essential information on scale structure, scoring, and thresholds. This revised version presents a concise overview suitable for a Methods section, focusing on how each instrument was applied in the study rather than describing each item in detail, in accordance with the reviewer’s recommendation.

***Comment 4***:

While the Vietnamese adaptation of MADRS is cited, the validation status of the other scales (HAM-A, ISI, ASEX) for this population is not reported. This should be added.

***Response:***

Thank you for pointing this out. We have now clarified the validation status of all instruments and described how measurement reliability and basic construct validity were evaluated in the present cohort. Only the MADRS had previously undergone full cultural adaptation and psychometric validation in Vietnamese patients with RA, as reported in an independent study by our group. The remaining scales—HAM-A, ISI, ASEX, and EQ-5D-5L—had not been validated in Vietnamese RA populations prior to this study. To address this gap and to establish reliability within the present cohort, we conducted a preliminary psychometric assessment of all remaining instruments. All scales were translated using the same forward–backward translation, harmonization, and pilot-tested face validity procedures described in the MADRS validation study. In this cohort, we calculated Cronbach’s alpha for internal consistency and performed confirmatory factor analysis (CFA) when conceptually appropriate. Additional measurements—item–item correlation and item–total correlation—were also examined.

The resulting internal consistency coefficients and validity indicators have been added to Supplementary Table S1. In summary:

- HAM-A and ISI showed high internal consistency and acceptable correlation patterns; their CFA models demonstrated borderline but interpretable fit.
- ASEX showed good internal consistency, but CFA was not feasible due to the very small number of participants reporting sexual activity.
- EQ-5D-5L demonstrated moderate internal consistency and coherent item–score correlations; factorial validation was not applicable because the instrument is formative.

All corresponding text has been added to the revised Study Outcomes section, and the full preliminary validation results are reported in Table S1.

***Comment 5***:

The rationale for using SEM should be elaborated (why this model was selected over simpler correlation/regression frameworks). Clarify fit indices used to validate SEM and whether assumptions (normality, multicollinearity) were verified.

***Response:***

Thank you for this important comment. We have expanded the Methods section to clarify the conceptual rationale for using structural equation modeling (SEM) and to report model diagnostics. SEM was selected because our objective was not only to examine pairwise correlations but to characterize the broader structure of psychiatric and behavioral disturbances in RA. Conceptually, we hypothesized that depression and anxiety (psychological symptoms) may cluster together to present a broader latent concept, while insomnia and sexual dysfunction represent behavioral manifestations that are closely intertwined with psychological distress. SEM allows these constructs to be simultaneously modeled as latent factors and permits estimation of the strength of their interrelationships, thereby providing a more integrated depiction of the mental health profile in RA. This approach offers insight into whether the presence of one disturbance implies elevated risk for others, which is clinically relevant for designing comprehensive mental health screening strategies in rheumatology practice. We have now clearly reported the SEM fit indices used to evaluate model adequacy, including χ², CFI, TLI, RMSEA (with confidence intervals), and SRMR. Model assumptions were also assessed: all observed variables demonstrated acceptable skewness and kurtosis under MLR estimation, and multicollinearity diagnostics (VIF range: 1.89–3.35) indicated no collinearity concerns. No model modifications were required, as all modification indices were low. These results are now summarized in Supplementary Table S2, which includes standardized loadings, R² values, fit indices, and diagnostic statistics.

Revisions made in the manuscript:

- Methods subsection “Statistical analysis” expanded to explain the rationale for SEM and the conceptual grouping of psychiatric vs. behavioral symptoms.
- SEM fit indices and assumption checks added.
- Supplementary Table S2 created to summarize SEM results.

***Comment 6***:

It would be helpful if the authors could support the choice of using SEM for such a limited sample size with an appropriate reference. It would be useful for the authors to clarify whether it is appropriate to perform SEM with a relatively small sample size, and to support this choice with a reference.

***Response:***

We appreciate the reviewer’s concern regarding the sample size for SEM. Although larger samples are generally preferable, methodological studies indicate that SEM can be adequately estimated with samples around 100–200 in models of low complexity (Wolf et al., 2013 – PMID: 25705052; Deng, 2018 - PMID: 29755388). Given that our SEM includes only two latent variables and four indicators, our sample of 187 is consistent with these recommendations, supporting the appropriateness of the analysis in this cohort.

***Comment 7***:

The results section largely repeats data from the tables, which could be streamlined. Key numerical findings should be emphasized selectively rather than listed exhaustively.

***Response:***

We thank the reviewer for this suggestion. In response, we have streamlined the Results section to emphasize key findings while avoiding exhaustive repetition of data already presented in tables. Detailed prevalence percentages for each severity category are retained in Tables 1–3, allowing readers to access complete data as needed, while the text now highlights the most clinically and statistically relevant outcomes.

***Comment 8***:

Significance levels (p-values) are not reported in the text for the main associations, limiting interpretability; these should be added where relevant.

***Response:***

P-values have been added for key associations, enhancing interpretability of the results.

***Comment 9***:

Odds ratios are sometimes large with wide confidence intervals (e.g., OR 11.2, 95% CI 3.07–57.9). The authors should discuss the potential for overfitting due to limited events (n=25 suicidal ideation cases).

***Response:***

As suggested, we have included a discussion of this limitation in the Discussion section, highlighting that the small number of suicidal ideation events (n = 25) may lead to imprecise estimates. We emphasize that these findings should be interpreted cautiously, although they still provide important insights into potential predictors of suicidal ideation in RA.

***Comment 10***:

Table 2 is difficult to read and interpret due to its dense structure; it should be graphically simplified or reorganized to improve clarity and readability.

***Response:***

We thank the reviewer for the suggestion to improve readability of Table 2 by using graphical formats. We agree that visual representation can often enhance interpretability. However, in this study, each psychometric instrument reports outcomes in a different format: depression, anxiety, and insomnia are classified into ordinal severity categories; sexual dysfunction and suicidal ideation are binary (yes/no) variables; and quality of life (EQ-5D-5L) is a continuous score. Attempting to combine these heterogeneous data into a single figure would result in a cluttered and confusing visualization, making it difficult for readers to accurately interpret each measure. Therefore, we have retained Table 2 in its current format, which presents the complete information clearly and allows readers to see both absolute counts and percentages (or mean ± SD for EQ-5D-5L) for all variables. We believe this approach balances comprehensiveness with clarity and ensures that all relevant data are accessible.

***Comment 11***:

The description of SEM (Figure 2) lacks goodness-of-fit statistics (CFI, RMSEA, etc.). Include these metrics to strengthen credibility.

***Response:***

We appreciate the reviewer’s comment regarding the inclusion of SEM goodness-of-fit indices. In response, we have provided a detailed summary of all relevant SEM statistics—including CFI, TLI, RMSEA, and standardized loadings—in Supplementary Table S2. To avoid redundancy and maintain clarity in the main Results section, we have focused on the key substantive findings of the SEM model: namely, that depression (DEP) and anxiety (ANX) loaded strongly onto the latent factor PSY, sexual dysfunction (SEX) and insomnia (SLP) loaded onto BEHAV, and the correlation between PSY and BEHAV was high (r = 0.88), indicating substantial overlap between psychological and behavioral disturbances. Presenting the fit indices only in the supplementary table ensures completeness for interested readers without overcomplicating the main narrative.

***Comment 11***:

Deepen analysis of causality limitations inherent in the cross-sectional design. The section currently acknowledges this but does not discuss how it affects the interpretation of associations.

***Response:***

We thank the reviewer for highlighting the importance of clarifying the implications of the cross-sectional design for causal inference. While we have acknowledged this limitation in the Discussion, we have now expanded the explanation to specify how it affects the interpretation of our findings. Because all variables—including disease activity, treatment-related adverse effects, and psychiatric disturbances—were measured at a single time point, we cannot establish temporal precedence or determine whether one factor directly causes another. For example, although high disease activity was strongly associated with suicidal ideation, it remains possible that pre-existing depression or anxiety could contribute to perceived disease severity or reduced adherence, creating bidirectional effects. Similarly, associations between adverse drug events and psychiatric outcomes may reflect both causal and reverse pathways. Consequently, all reported associations should be interpreted as correlations rather than evidence of direct causality. We have also emphasized that longitudinal studies are necessary to clarify the directionality and temporal dynamics of these relationships, which would better inform early intervention strategies and risk stratification in patients with RA.

***Comment 12***:

Discuss cultural or health system factors that may influence mental health disclosure or underreporting in the Vietnamese context.

***Response:***

We appreciate the reviewer’s comment highlighting the potential influence of cultural and health system factors on mental health reporting in Vietnam. We have added a discussion in the limitations section to acknowledge that mental health stigma and social norms emphasizing resilience may discourage patients from openly disclosing depressive symptoms, anxiety, or suicidal thoughts. Consequently, self-reported or interview-based assessments could be affected by social desirability bias, potentially leading to underestimation of the prevalence and severity of psychiatric disturbances. We also note that limited integration of mental health services within rheumatology care may further reduce opportunities for disclosure. This discussion emphasizes the importance of culturally sensitive and systematic mental health screening in future studies to ensure more accurate assessment of psychological burden.

***Comment 13***:

Consider contrasting the current prevalence with international cohorts using similar tools (rather than citing only one Mexican study).

***Response:***

We sincerely thank the reviewer for this suggestion. We conducted an additional literature search focusing specifically on international rheumatoid arthritis (RA) cohorts that directly reported the prevalence of suicidal ideation using comparable assessment methods. However, to the best of our knowledge, beyond the 2022 Mexican outpatient cohort, no other published RA studies have reported suicidal ideation as an independent outcome. Existing RA literature predominantly examines depression and anxiety, whereas suicidal ideation has rarely been measured directly or reported as a standalone variable. Given the lack of comparable RA-specific cohorts, we expanded the Discussion to provide a broader contextual comparison by summarizing suicidal ideation rates from other autoimmune and chronic inflammatory diseases, including systemic lupus erythematosus, fibromyalgia, and multiple sclerosis. These conditions share similar immunological and psychosocial burdens with RA and therefore offer a meaningful background for interpreting the magnitude of suicidality seen in our study. This revision clarifies the current evidence gap within RA research and situates our findings within a wider psychoneuroimmunology framework. The corresponding changes have been added to the Discussion section.

***Comment 14***:

Reorganize to separate interpretive content (mechanisms, implications) from methodological limitations to improve flow.

***Response:***

We appreciate the reviewer’s suggestion regarding the organization of the Discussion. In the current version, interpretive content (mechanisms, clinical implications, and contextualization with prior literature) is already grouped together in the first several paragraphs, whereas methodological limitations are fully consolidated in the final paragraph of the Discussion. This structure follows the conventional format used in rheumatology and psychiatric epidemiology manuscripts.

Nonetheless, we have carefully reviewed the section to ensure clear separation between interpretation and limitations, and we confirm that the flow is coherent and consistent with the reviewer’s recommendation.

***Comment 15***:

Improve transitions between paragraphs in the Discussion to reduce repetition of similar concepts (particularly the link between disease activity and psychological morbidity).

***Response:***

We appreciate the reviewer’s insightful comment. We have carefully reviewed the Discussion and refined transitions between paragraphs to improve flow. Redundancies—particularly those related to the link between disease activity and psychological morbidity—have been minimized, and several bridging sentences were added to ensure smoother progression of ideas. These revisions enhance readability without altering the core interpretation of our findings.

***Comment 16***:

The “Data collection” subsection should be repositioned between “Participants” and “Study outcomes” to improve logical flow and coherence, ensuring that the description of procedures follows directly after the participant characteristics and before the outcome definitions.

***Response:***

We thank the reviewer for this helpful suggestion. The “Data collection” subsection has now been repositioned between “Participants” and “Study outcomes” to enhance logical flow and improve the coherence of the Methods section. This restructuring ensures that procedural details follow directly after participant characteristics and precede the description of study outcomes, as recommended.

***Comment 17***:

There are some errors and redundant symbols in Table 1. For example, it would be necessary to remove “n (%)” next to each variable and include in the header the wording “Variable, n (%)”. It would also be appropriate to remove the “%” symbol inside the parentheses of the frequencies. Moreover, typographical errors include “suicial ideation” (page 8), “Collegue” (Table 1), “Marrital” (Table 1), and “Halminton” (Table 2).

***Response:***

The term “suicial ideation” has been corrected to “suicidal ideation.” In Table 1, the item originally written as “Collegue and above” contained a spelling error; it has now been corrected to “College and above”, which accurately reflects the intended educational category. “Marrital” has been corrected to “Marital” in Table 1, and “Halminton” has been corrected to “Hamilton” in Table 2. Regarding table formatting, placing “Variable, n (%)” in the header is not feasible because Table 1 includes both categorical variables presented as n (%) and continuous variables presented as mean ± SD. Therefore, the notation for categorical variables has been retained on a per-variable basis to avoid misrepresentation.

***Comment 18***:

Ensure consistent use of units and symbols (e.g., replace “X±SD” with “Mean ± SD”).

***Response:***

The notation for continuous variables has been standardized throughout the manuscript and tables. All instances of “X ± SD” have been replaced with “Mean ± SD” to ensure consistency and clarity.

***Comment 19***:

4.Replace overly technical or redundant phrasing with concise alternatives (e.g., “participants were informed about the study objectives and their right to withdraw” could be shortened to “participants provided written informed consent”).

***Response:***

Several instances of overly technical or redundant phrasing have been revised to more concise alternatives throughout the manuscript, including in the Methods section. These changes improve clarity and readability without altering the scientific content.

***Comment 20***:

Maintain consistent tense (past tense for methods and results).

***Response:***

The Methods and Results sections have been carefully reviewed, and all remaining instances of present-tense wording have been revised to the past tense to ensure consistency throughout the manuscript.

***Comment 21***:

Overall, review English phrasing for fluency and minor grammatical adjustments.

***Response:***

The entire manuscript has been carefully reviewed, and minor grammatical and phrasing adjustments have been made to improve clarity, accuracy, and overall fluency.

**Reviewer 3**

***Comment 1***:

The Introduction could be strengthened and expanded from a psychological and theoretical perspective. At present, it mainly focuses on the epidemiological and clinical burden of RA, but provides limited discussion of the psychological mechanisms linking chronic illness with suicidality. The section would benefit from a more developed framework describing how psychological and psychosocial processes contribute to suicidal ideation. Integrating theoretical perspectives (e.g., the hopelessness theory of depression or models addressing the burden and loss of meaning in chronic illness) would help situate the study more firmly within clinical psychology.

***Response:***

We appreciate the reviewer’s insightful suggestion. In the revised manuscript, the Introduction has been expanded to incorporate a clearer psychological and theoretical foundation. Specifically, we added (i) a brief explanation of the hopelessness theory of depression to illustrate how persistent, uncontrollable stressors in chronic illness may lead to negative future expectations and increased susceptibility to suicidal ideation, and (ii) elements from contemporary models of chronic illness adjustment, highlighting perceived burden, loss of autonomy, and disruptions in social roles and meaning-making. These additions provide a more comprehensive psychological framework linking RA-related stressors with suicidality and situate the study more firmly within relevant clinical psychology theories.

***Comment 2***:

While the Vietnamese version of the MADRS has been validated, the cultural adaptation or validation of the other measures (HAM-A, ISI, ASEX, EQ-5D-5L) is not discussed. Providing even a brief comment on their psychometric adequacy in this context would strengthen the study’s credibility.

***Response:***

Thank you for this helpful comment. We have now clarified the validation status of all psychiatric and QoL instruments. As noted, only the MADRS had undergone full cultural adaptation and psychometric validation in Vietnamese RA patients in a previous study by our group. The remaining scales (HAM-A, ISI, ASEX, EQ-5D-5L) had not been specifically validated in Vietnamese RA populations; therefore, we conducted a brief psychometric evaluation within the present cohort. All instruments were translated using the same forward–backward and harmonization procedures applied in the MADRS validation study. Internal consistency (Cronbach’s alpha), item–item and item–total correlations, and confirmatory factor analysis (when conceptually appropriate) were assessed. Overall, the HAM-A and ISI demonstrated high internal consistency; the ASEX showed acceptable reliability but CFA was limited by low sexual activity reporting; and the EQ-5D-5L exhibited moderate internal consistency, consistent with its formative structure. These results have been added to the Study Outcomes section and summarized in Supplementary Table S1.

***Comment 3***:

Another area for improvement concerns the literature review. The discussion relies heavily on a single reference for suicidal ideation in RA. Expanding this section to include findings from other chronic or autoimmune diseases (e.g., lupus, fibromyalgia, multiple sclerosis) would give a broader comparative perspective and help clarify the research gap that this study aims to fill. This would also position the work more clearly within the wider field of psychoneuroimmunology and chronic disease psychology.

***Response:***

We appreciate the reviewer’s insightful suggestion. As recommended, we have substantially expanded the literature review and Discussion to incorporate suicidal ideation data from other chronic autoimmune and inflammatory diseases, including systemic lupus erythematosus, fibromyalgia, and multiple sclerosis. These conditions share immunological, neuropsychiatric, and chronic pain mechanisms with rheumatoid arthritis and therefore provide valuable comparative context. This addition helps illustrate that suicidality is a recognized concern across immune-mediated diseases while simultaneously highlighting that RA-specific evidence remains notably sparse, with only one published cohort reporting suicidal ideation as an independent outcome. By integrating findings from related disease populations, the revised Discussion offers a broader comparative perspective and more clearly delineates the research gap that our study addresses. This framing also situates our work within the wider field of psychoneuroimmunology and chronic disease psychology, as the reviewer suggested. The manuscript has been revised accordingly.

***Comment 4***:

Finally, it would be appropriate to expand the limitations section by adding some methodological and psychological considerations that could influence the interpretation of the results.

Specifically, the assessment of suicidal ideation relied solely on clinical interviews without the inclusion of standardized instruments (e.g., C-SSRS), which may limit comparability across studies and introduce rater-dependent bias.

Given the potential stigma associated with mental illness and suicidality in Vietnam, self-reported or interview-based assessments may also have been influenced by social desirability bias, possibly leading to underreporting of psychological symptoms.

Moreover, the study did not account for potentially relevant cognitive or psychosocial variables (e.g., coping styles, perceived social support, or self-efficacy) that may act as mediators or moderators in the relationship between disease activity and mental health outcomes. Considering these factors in future research would enhance the psychological depth and explanatory power of the findings.

***Response:***

Thank you for these insightful suggestions. We have expanded the Limitations section to address each of these concerns. Specifically, we now:

1. Clarify the limitation of using clinician interviews without standardized instruments such as the C-SSRS, acknowledging potential rater-dependent variability and reduced comparability across studies.
2. Discuss the influence of cultural stigma and social desirability bias in Vietnam, which may contribute to underreporting of suicidal ideation and other psychiatric symptoms.
3. Acknowledge the absence of key psychosocial constructs (e.g., coping strategies, perceived social support, self-efficacy) that may mediate or moderate relationships between disease activity and mental health outcomes, and emphasize their importance for future research.

These additions have been incorporated into the revised Limitations section to provide a more complete and psychologically informed interpretation of our findings.
